# The role of vitamin C on the skin

**DOI:** 10.4102/safp.v67i1.6098

**Published:** 2025-07-14

**Authors:** Lehlohonolo Makhakhe

**Affiliations:** 1 Department of Dermatology, Faculty of Health Science, University of the Free State, Bloemfontein, South Africa; 2 The South African Institute of Dermatology, Bloemfontein, South Africa

**Keywords:** ascorbic acid, vitamin C, vitamin E, ageing, antioxidant, diet, skin application, UV radiation

## Abstract

Vitamin C (ascorbic acid or ascorbate) plays an important role in regulating the health of the skin, promoting the differentiation of epithelial skin cells (keratinocytes) while contributing a notable role in the reduction of melanin synthesis, leading to antioxidative protection against solar-related skin damage. Vitamin C is only sourced from diet because humans have no ability to synthesise it in vivo. Routine dietary intake choices become key in maintaining the skin’s integrity, preventing and treating some of the skin conditions encountered regularly by general practitioners and skin specialists alike. There is a strong association between vitamin C and the ageing process, skin pigmentation, control of certain skin diseases and a role in some skin cancers through antioxidation properties. Literature suggests that topical application of vitamin C in different forms of formulations has been demonstrated to have more clinical effect than oral intake.

**Contribution:**

This article spotlights the benefits of a vitamin commonly encountered in topical pharmaceutics, ingested as tablets or as part of our routine diet.

## Introduction

The skin is complex and multi-functional; being the largest organ of the body and easily clinically accessible, it can further serve as a reflective source of internal health of the rest of the body. The skin’s fundamental role is in providing a protection shield against external environmental threats, such as microbes, chemicals, allergens and radiation.^1^

**FIGURE 1:**
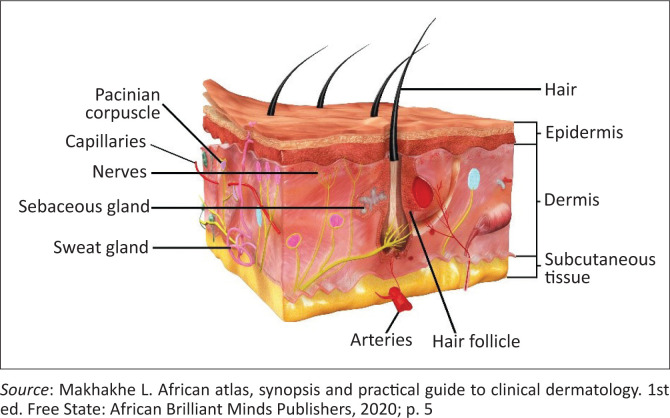
Skin anatomy.

In different cutaneous layers, vitamin C plays a pivotal role in aiding the skin to carry out some of the key functions. These vary from preventative, protective, healing and rejuvenative to assistance in the clinical improvement of some dermatological conditions.^2^ The epidermis primarily houses keratinocytes and melanocytes, where the antioxidative effects of vitamin C are mostly evident. The dermal layer of the skin provides strength and elasticity and further houses the neurovascular structures, glands and a network of collagen fibres^3^ (Figure 1).^4^ There is a marked difference in the levels of vitamin C in the different layers of the skin. The concentration of ascorbic acid in the epidermis is 425% higher than in the dermis.^5,6^ In the modern era, an accurate method of measuring vitamin C levels within the skin entails taking a skin biopsy. The sample is then analysed utilising high-performance liquid chromatography (HPLC).^7^ Within the epidermal keratinocytes, there is a concentration gradient of ascorbic acid with a sharp increase in concentration in the deeper layers of the stratum corneum, possibly reflecting depletion in the outer cells attributable to chronic exposure to the external environment. Literature also suggests significant uptake from the gut, rather than cutaneous application, as a source.^8,9^

Several reports have indicated that vitamin C levels are lower in chronologically aged and photo-damaged skin. Chronological ageing is defined as a natural ageing process through cumulated years, and photo-damage is because of accelerated skin ageing from chronic sun exposure. Whether the low levels of ascorbic acid reflect the cause or effect of ageing remains unclear to date; consequently, this warrants further research in this regard.^1^

The pharmaceutical industry has a variety of topical formulations with vitamin C contained; these predominantly include creams and gels.

In this article, we review the common physiological benefits of vitamin C on the skin, give an overview of related properties, the role of this vitamin in skin health and disease, homeostasis and the synergistic role with other vitamins, such as vitamin E (tocopherol).

To conduct this review, we utilised the search engines Google Scholar and PubMed, employing specific keywords to search for any relevant articles written in English from 1994 to 2024. A total of 204 articles were identified, with duplicates removed, resulting in 129 articles reviewed. Of the remaining articles, just over 80 were selected, with greater emphasis placed on the more recent publications because of the rapid pace of new developments in knowledge regarding this topic.

## Discussion

### The role of vitamin C in the prevention of skin ageing

In broad terms, skin ageing can be attributed to several factors, including, but not limited to, chronological and photoageing, smoking, chronic alcohol usage, chronic stress, some medical conditions and a lack of self-care. Normal (intrinsic) ageing contributes directly to loss of elasticity and wrinkle formation; it is largely an unavoidable process that can also be directly linked to genetics.^2,10^

Within the epidermis, lack of vitamin C can result in peroxidative damage to skin cells; furthermore, the epidermis is constantly exposed to ultraviolet (UV) radiation, further resulting in accelerated ageing because of reactive oxygen species (ROS). In the dermis, deficiencies in ascorbic acid can cause damage to the connective tissue structures, also because of peroxidative stress. Damage can also be as a result of ROS owing to the rich dermal blood supply. While it remains unclear whether high dietary vitamin C intake has better long-term benefits, research suggests that lack of it results in increased oxidative damage, especially in the presence of continuous UV exposure, thus leading to skin ageing.^11,12,13^

Intrinsic ageing tends to be a protracted natural process, and in the absence of other aggravating factors, changes are not usually apparent until advanced age is reached. The skin changes are typified by fine wrinkling, reduced elasticity, skin atrophy and occasional exaggeration of the Langer’s lines expression may be evident. These skin signs may be seen independently, or they may be present in an overlapping sequence.^14,15^

As ageing progresses, there is a notable decline in the recoiling and elastic capacity of the skin; this is in direct relation with a collective number of factors, such as reduced fibrovascular support structure and a marked decline in extracellular matrix components like collagen and elastin.^16,17^

The ageing process also results in dry skin (xerosis) because of a gradual decline in glycosaminoglycans within the dermis; however, they help in forming part of the structural network and in retaining dermal moisture. In addition to the reduced ability to retain moisture, skin fragility and easy bruising become progressively apparent as ageing advances, more so in sun-exposed areas such as the face.^18,19^

The aged epidermis demonstrates a reduced barrier function and reduced repair properties following traumatic insults. In general, the dermis suffers greater age-related changes than the epidermis. Antioxidant capacity, immune regulation and melanin production appear to also be impaired in aged skin.^20,21^

The regular use of ascorbic acid-containing supplements, either orally or topically, has the potential to reverse skin ageing by regenerating the elastic fibre system and collagen within the dermis.^22^

### Role in hyperpigmentation and skin lightening

Vitamin C has been shown to decrease melanin synthesis by inhibiting melanogenesis both in cultured melanocytes and *in vivo*.^23^

This is believed to be because of its ability to interfere with the action of tyrosinase, a rate-limiting enzyme in melanogenesis. Agents that decrease melanogenesis are used to treat skin hyperpigmentation in conditions such as melasma and post-inflammatory hyperpigmentation from different causes. These largely include hydroquinone, glutathione, azelaic acid, niacinamide, glycolic acid, kojic acid, vitamin E, retinoids and cannabidiol.^24,25^

Skin lightening treatments are steadily gaining traction in the beauty industry, two of the most common reasons being to treat widespread forms of non-segmental vitiligo and for those who find light skin complexion to be of superior appeal. A combination of intravenous glutathione and vitamin C is the popular choice for total eradication of the melanin pigmentation.^26,27^

### Vitamin C and wound healing

Vitamin C acts as a cofactor for the amino acids proline and lysine. It stabilises collagen through hydroxylation, and it also promotes collagen gene expression. Vitamin C may prevent collagen degradation and inhibit the increase of matrix metalloproteinase-1 (MMP-1), which is the major collagenolytic enzyme responsible for collagen damage in UV-irradiated skin.^28,29^

Wound healing is generally characterised by three overlapping stages, namely inflammation, re-epithelialisation (new tissue formation) and remodelling. The inflammatory stage entails neutrophils as the first cells to the site to engulf damaged tissue and debris and signal macrophages to continue with the consumption of the debris, including spent neutrophils. Various cytokines continue onwards, coupled with fibrin and fibroblast proliferation as re-epithelialisation and remodelling takes place, mostly with scar tissue to a varying degree. Re-epithelialisation restores the skin’s barrier function and occurs by a combination of migration and proliferation of the epidermal keratinocytes around the wound site.^30,31,32^

Vitamin C has been shown to regulate elastin synthesis in cultured fibroblasts and has a marked increase in turnover at the wound sites, because of both local inflammation and the demands of increased collagen production, supporting theories of supplementation being beneficial both in topical and oral intake. Studies have shown that supplementation with both vitamin C and vitamin E improved the rate of wound healing in individuals with extensive burns.^33,34^

### Vitamin C and nutrition

From a nutritional perspective, vitamin C is water soluble, heat sensitive and easily oxidised. Refrigeration aids in retaining its nutritional value. Fruit and vegetable sources should be eaten unpeeled if possible and not be cut into smaller pieces to avoid exposing cells to oxidation. Soaking in water is also discouraged, along with cooking with excessive water or a poorly fitting pot lid. The best sources of vitamin C include citrus fruit, orange juice, spinach, cabbage, green pepper, Brussels sprouts, turnips, guavas, pineapples, sweet melons, fresh strawberries and correctly prepared potatoes. Smaller quantities of this vitamin can also be found in peaches, pears, apples, bananas and blueberries.^35,36^

Adequate vitamin C as part of a routine diet has been linked with enhanced skin barrier, thereby improving dry skin and smooth skin surface. Vitamin C concentration within the keratinocytes can double upon supplementation with certain foods or 3 g/day supplementation for 6 weeks, a dosage that is significantly higher than the recommended daily intake and would achieve plasma saturation and likely also tissue saturation.^37,38^

### Vitamin C deficiency

Nutritional manifestations are largely in the form of insufficiency states. Scurvy is the clinical manifestation of vitamin C deficiency. Because vitamin C plays a central role in the protective functions of the skin, nutritional deficiency results in skin fragility, perifollicular petechiae, ecchymosis (easy bruising), gingivitis, corkscrew hairs and poor wound healing. Causes may include chronic alcohol abuse, chronic smoking, mental care users, patients on chronic dialysis and poor intake for a plethora of possible reasons.^12,39,40^

### Promoting differentiation of keratinocytes

Through the process of differentiation, keratinocytes play a key role in skin barrier function, thus preventing water loss and microbial invasion to deeper layers. Vitamin C plays an important role in aiding with differentiation.^41^ Patients with atopic dermatitis have been reported to also suffer from food allergens as a potential trigger source. There is a plausible association between vitamin C deficiency and atopic eczema, resulting from certain food avoidance and precautionary food consumption.^42,43,44,45^

Furthermore, vitamin C can stimulate ceramide production within keratinocytes and improve overall epidermal barrier function. With increases in clinical symptoms of atopic dermatitis, vitamin C and ceramide levels were reduced; however, this suggests a positive correlation between this form of dermatitis, vitamin C and the natural skin lipids.^46,47^

### Melanoma

The toxic effects of vitamin C on tumour cells may be related to the induction of oxidative stress in cells. Vitamin C can also increase 5-hydroxymethylcytosine (5-hmC) content in melanoma, and this has been demonstrated to reduce tumour growth.^48,49^

Moreover, cancer patients have been shown to have very low reserves of ascorbic acid, which is essential for the structural integrity of the intercellular matrix, as already alluded to. Degradation of the extracellular matrix correlates with the aggressiveness of tumour growth and invasiveness of a cancer. As part of adjuvant therapy, ascorbic acid supplementation has been found to significantly reduce the metastasis of melanoma in mice. Vitamin C can reduce tumour growth, invasion and metastasis of melanoma in mice by inhibiting the hypoxia-inducible factor-1 alpha (HIF-1α) transcriptional activity, which might play a key role in melanoma carcinogenesis.^50,51^

### Epigenetic pathways

In addition to the gene regulatory activities, vitamin C has a role in epigenetic regulation of gene expression by functioning as a cofactor for the ten-eleven translocation (TET) family of enzymes, which catalyse the removal of methylated cytosine through its hydroxylation to 5-hydroxymethylcytosine.^52^

Because TETs have a specific requirement for vitamin C to maintain enzyme activity, this provides a further mechanism by which the vitamin may affect gene expression and cell function.^53^

Aberrant epigenetic alterations are thought to have a role in cancer progression, and there are data to suggest that a loss of 5 hmC occurs during the early development and progression of melanoma.^54^

### Synergistic effects of vitamin C, vitamin E and selenium

The combination of vitamin E and vitamin C has been shown to reduce the incidence of oxidative stress-induced tumours, and their antioxidant effects are much better than the use of vitamin C alone.^70,71^ This synergy holds true also when ferulic acid and selenium are added as well.^3^

The combination of vitamin C and vitamin E inhibits melanocyte production more significantly than vitamin C alone.^6^

Research has also shown that the combination of vitamin A, vitamin C and zinc leads to quicker recovery in wound healing. This is also demonstrated in venous ulcers, as vitamin C stimulates fibroblast proliferation while vitamin A and zinc assist greatly in immune-related functions.^72^

In addition, vitamin C directly affects the immune system to reduce the chance of viral infection in the body, similar to the application of vitamin D, which can affect the immune mechanisms of the human body. Therefore, whether the combined use of vitamin C and vitamin D has a good and comprehensive therapeutic effect on the presence of herpes zoster and post-herpetic neuralgia is still a question worth exploring.^73,74^

The role of ascorbic acid is also noted in other dermatology-related skin conditions, see Table 1.

**TABLE 1:** The role of vitamin C in other skin diseases.

Skin conditions	Association with vitamin C
SLE	Vitamin C supplementation can have beneficial effects on patients with SLE.^55,56^
Non-melanoma skin cancers	UV radiation has the potential to damage hydrogen bonds between DNA double strands and induce oxidative stress. Oxidative stress can further lead to alteration of nucleic acids within affected cells, resulting in tumour growth. Antioxidative properties of vitamin C can thus play a significant role in the adjuvant treatment of non-melanoma carcinomas (photocarcinogenesis).^19,57,58^
PCT	The pathophysiology of PCT is predominantly related to the iron content: the greater the iron load, the more severe the disease.^59^ As an antioxidant, ascorbic acid has the potential to inhibit the catalytic oxidation of the enzyme CYP1LA2, which is known to help in the production of uroporphyrin and heptacarboxylporphyrin.^60^ However, caution must be observed as ascorbic acid promotes iron absorption in the intestine, which may be risky in patients with iron overload, thus worsening the overall clinical picture. There is room for further research in this regard.^61^
Keloids and other types of scarring	Vitamin C supplementation can assist in preventing keloid and scar formation through fibroblast regulation and antioxidant properties.^62,63^
Acne vulgaris	Vitamin C has an anti-inflammatory effect because of its ability to inhibit NF-kB, which is responsible for the activation of inflammatory cytokines including IL-1, IL-6, IL-8 and TNF-alpha. Furthermore, its wound healing and anti-hyperpigmentation properties as well as antioxidant properties are important in the skin care for acne vulgaris patients.^64^
Herpes zoster and post-herpetic neuralgia	Vitamin C, as an oxidant, has been reported to have a clinical analgesic effect. In addition, the incidence of post-herpetic neuralgia in patients with herpes zoster who lack plasma vitamin C has been significantly higher than the incidence in patients with normal plasma vitamin C levels. When vitamin C supplementation is given to patients with herpes zoster, the probability of subsequent neuralgia can be reduced.^65,66^
Genital herpes	Vitamin C leads to improved immunity and natural defences while reducing the persistence of herpes infection.^67^
Vitiligo	Studies have found that patients on vitamin C supplementation have increased peri-follicular re-pigmentation.^68^
PPPD	Ascorbic acid promotes the protection of blood vessel collagen, reduces vascular fragility and prevents disease recurrence.^69^

*Source:* Wang K, Jiang H, Li W, Qiang M, Dong T, Li H. Role of vitamin C in skin diseases. Front Physiol. 2018;9:819. https://doi.org/10.3389/fphys.2018.00819

SLE, systemic lupus erythematosus; UV, ultraviolet; DNA, deoxyribonucleic acid; PCT, porphyria cutanea tarda; NF-kB, nuclear factor kappa B; IL, interleukin; TNF-alpha, tumour necrosis factor-alpha; PPPD, progressive pigmented purpuric dermatosis.

### Topical application

Vitamin C is a charged molecule that is repelled by the physical barrier of the terminally differentiated epidermal cells. Transcutaneous penetration occurs when the pH levels are below 4; this is when vitamin C is present as ascorbic acid, but whether this results in increased levels in the metabolically compromised stratum corneum is unknown. Furthermore, absorptive capacity also depends on the pH of the formulation that vitamin C is placed in.^3,75^

Vitamin C can be delivered to the epidermal layer by topical application although the efficacy of this is dependent on the formulation of the cream or serum used on the skin. This entails gentle rubbing of the vitamin, and in this regard, available literature leans more towards the cosmetic value of topical applications containing this vitamin, predominantly in anti-ageing, treatment of hyperpigmentation and skin lightening.

The main focus in current research is on the development of ascorbic acid derivatives for the purpose of topical application. Such derivatives need to ensure stabilisation of the molecule from oxidation and overcome the significant challenge of skin penetration. In addition, they must be converted to ascorbic acid *in vivo* to be effective. The addition of a phosphate group confers greater stability yet poor epidermal absorption.

### Oral intake

Vitamin C is an affordable and available over-the-counter vitamin with low toxicity potential; this aids immensely in exploring all potential medicinal benefits. Oral intake depends on the objective because this vitamin is associated with a wide range of medicinal benefits; the duration, frequency and dosing largely depend on the desired outcome. With adequate dietary intake, oral supplementation has been found to be of limited value.^12^

There are no studies that have investigated the relationship between skin vitamin C content in relation to oral supply, be it from food or supplementation, when plasma levels are low. However, clinical improvement has been noted in the absence of active measuring of the cutaneous levels. Serum levels are also seldom measured, even in cases of proven clinical deficiencies in developing countries.^3^

For scurvy, a dosage of 100 mg – 500 mg per day for 2 weeks can result in satisfactory clinical outcomes but must be followed up with a review, and the cause of the deficiency must be identified. Low doses contained within a multivitamin supplementation tablet can be considered as part of health supplementation and optimised immunity. Larger studies are needed to evaluate the optimal formulation, dose, frequency and duration in treating other skin conditions as part of the adjuvant therapy.^76^

### Intravenous intake

This is favoured when used in synergy with glutathione when aiming to universally lighten the skin for those lured by the cosmetic appeal. The appeal may be in those who perceive darker skin as unsatisfactory or those with widespread non-segmental vitiligo. This appeal is predominantly seen among the female gender.^77^

### Toxicity

The recommended daily oral amount for vitamin C is 75 mg a day for women and 90 mg a day for men. During pregnancy, 120 mg a day is recommended because of higher demands of the gestational period. The upper limit for all adults is 2000 mg a day. There is scant and inconclusive data with regard to vitamin C toxicity or complications thereof.^78^

Topical vitamin C is available in the market as a variety of creams, sera and transdermal patches. Of these, only the serum contains active forms of this vitamin in an almost colourless form. It is unstable and, on exposure to light, gets oxidised to dehydroascorbic acid (DHAA), which imparts a yellow colour. Minor adverse reactions include a yellowish discolouration of the skin, hypopigmented hair and staining of clothes, which occur because of oxidative changes. Once applied, it cannot be fully washed or wiped off the skin. Rarely, stinging, erythema and dryness are observed after topical usage. These can easily be treated using a moisturiser.^79,80,81,82^

## Conclusions

The ability to exclusively measure the efficacy of oral or topically applied vitamin C in preventing ageing remains practically challenging in the wake of other attributing factors.

There is limited evidence in the literature supporting a link between serum vitamin C levels and ageing changes. Vitamin C levels are also almost never measured in the skin, and this information is needed before we can improve our understanding of what level of intake might be beneficial for skin health and protection against ageing-related changes. With the increasing use of HPLC, the correlation between skin concentration and the general health of the skin becomes a key focus of future research output.

In conclusion, vitamin C has many plausible uses in medicine although further research is warranted with regard to dosages for different indications. In the main, past work has focused on the reasons why ascorbic acid can work through its properties and mechanisms of action clinically. Future research must be centred around dosage, frequency, duration, ideal formulation and in-depth monitoring of ideal cutaneous concentrations in relation to response to therapy or supplementation.
